# Endoscopic posterior cervical canal decompression versus laminoplasty for two-level cervical spondylotic myelopathy: clinical outcomes and finite element analysis

**DOI:** 10.1186/s13018-026-07000-1

**Published:** 2026-06-08

**Authors:** Bai Dong, Zhihua Yan, Xiao Sun, Jun Tan, Honggang Zhou, Mingkui Shen, Haijun Ma

**Affiliations:** 1 Department of Mini-invasive Spinal Surgery, The Third People’s Hospital of Henan Province, Zhengzhou, 450006 Henan China; 2 Henan Engineering Research Center of Precision Diagnosis and Treatment of Intervertebral Disc Disease, Zhengzhou, 450006 Henan China

**Keywords:** Endoscopic posterior cervical canal decompression, Laminoplasty, Cervical spondylotic myelopathy, Finite element analysis, Learning curve

## Abstract

**Objective:**

To comprehensively evaluate the clinical efficacy, surgical invasiveness, learning curve, and biomechanical characteristics between endoscopic posterior cervical canal decompression (Endo-PCCD) and laminoplasty (LP) in patients with two-level cervical spondylotic myelopathy (CSM). This study uniquely integrates clinical outcomes, cumulative sum (CUSUM) learning curve analysis, and finite element modeling to provide a multidimensional comparison between the two procedures.

**Methods:**

This retrospective study included 95 patients with CSM, of whom 45 underwent Endo-PCCD and 50 underwent LP. Baseline characteristics, perioperative parameters, and clinical outcomes (VAS, JOA, and NDI) were compared. DSA improvement rates were measured at C3-C5, C4-C6, and C5-C7 levels. Learning curves were assessed based on cumulative sum (CUSUM) analysis. Cervical finite element models were developed to evaluate the segmental range of motion (ROM) of Endo-PCCD and LP under identical loading conditions.

**Results:**

Baseline characteristics were comparable between groups. The Endo-PCCD group showed significantly shorter operative time, lower drainage volume, and higher postoperative hemoglobin (Hb) levels (all *P* < 0.05). Clinical outcomes were similar at all follow-up time points. DSA improvement was significantly greater in the LP group at C5-C7 (*P* < 0.001), with no differences at other levels. Proficiency thresholds were 25 cases for Endo-PCCD and 19 cases for LP. Finite element analysis indicated that the LP model exhibited increased segmental ROM under flexion and extension, whereas the Endo-PCCD model maintained motion patterns closer to the intact spine.

**Conclusion:**

Endo-PCCD achieves clinical outcomes comparable to LP while offering a potential trend toward reduced surgical invasiveness and may better preserve physiological cervical biomechanics, although LP may provide greater decompression at certain levels.

*Clinical trial number* Not applicable.

**Supplementary Information:**

The online version contains supplementary material available at 10.1186/s13018-026-07000-1.

## Introduction

Cervical spondylotic myelopathy (CSM) represents the most frequent cause of spinal cord dysfunction in adults [1]. It develops as a consequence of progressive degenerative changes affecting the cervical vertebrae, intervertebral discs, facet joints, and associated ligaments structures, which ultimately compress the spinal cord and nearby vascular tissues [2]. Clinically, degenerative cervical myelopathy is characterized by diverse manifestations, varying from mild sensory disturbances to quadriparesis accompanied by severe sphincter dysfunction [3]. Patients with minimal symptoms and without definite evidence of myelopathy are generally managed non-surgically, whereas patients with definitive myelopathic features and radiographic evidence of spinal cord compression are considered appropriate candidates for surgical intervention [4].

The surgical management of CSM mainly includes anterior, posterior, and combined approaches [5]. In recent years, with advances in endoscopic spinal techniques, increasing evidence suggests that percutaneous endoscopic cervical discectomy (PECD) can achieve clinical outcomes comparable to anterior cervical discectomy and fusion (ACDF), while offering perioperative benefits such as reduced operative time and shorter hospitalization [6]. Kim et al. further reported that biportal endoscopic posterior cervical laminectomy is a safe and effective option for multilevel CSM [7]. Moreover, traditional open cervical surgery often requires extensive paraspinal muscle dissection and bone removal, which may compromise spinal stability. Biomechanical studies have demonstrated that posterior cervical components, such as the spinous processes, supraspinous and interspinous ligaments, facet joints, and articular processes, play a critical role in maintaining cervical spinal stability [8]. In this context, endoscopic posterior cervical canal decompression (Endo-PCCD) has emerged as a promising minimally invasive technique. Compared with laminoplasty (LP), Endo-PCCD causes less soft tissue disruption and may better preserve posterior stabilizing structures. Nevertheless, evidence directly comparing the clinical efficacy of Endo-PCCD and LP for CSM remains limited.

Meanwhile, finite element analysis is widely applied to simulate and predict the biomechanical responses associated with different spinal surgical procedures [9]. Previous studies have demonstrated that imaging-based three-dimensional finite element models can reliably reproduce cervical spine biomechanics under physiological loading conditions and predict postoperative alterations in range of motion (ROM), stress distribution patterns, and stability status [10, 11]. However, most existing finite element studies have focused on anterior surgical approaches, cervical degenerative conditions, or fusion procedures [12, 13]. Biomechanical investigations of posterior decompression surgery, particularly systematic comparisons between Endo-PCCD and LP, remain relatively limited. Consequently, the effects of these two techniques on segmental ROM and cervical stability have not been fully elucidated.

Based on these considerations, we conducted a comparative study of Endo-PCCD and LP in patients with CSM. By integrating clinical outcomes and biomechanical evaluation, we sought to determine whether Endo-PCCD could achieve clinical efficacy comparable to LP while offering a potential trend toward reduced surgical invasiveness and maintaining more physiological cervical spine biomechanics.

## Materials and methods

### Study design

This is a retrospective case-control study. A total of 95 patients with CSM treated at our department between May 2023 and December 2024 were retrospectively analyzed, including 45 patients in the Endo-PCCD group and 50 in the LP group. The surgical procedure was determined through shared decision-making between patients and the treating surgical team, based on a comprehensive evaluation of clinical symptoms, imaging findings, and overall patient condition.

Written informed consent was obtained from all participants. The study received approval from the Ethics Committee of our hospital and was conducted in accordance with internationally accepted ethical standards, including the Declaration of Helsinki.

## Patient selection criteria

Patients were eligible for inclusion if they met the following conditions: (1) A diagnosis of two-level CSM confirmed by clinical symptoms, physical signs, and imaging findings; (2) Patients requiring surgical intervention due to failure of conservative treatment or progressive decline of spinal cord neurological function; (3) Patients mainly presenting with cervical spinal cord compression caused by posterior or posterolateral degenerative structures; (4) Patients without surgical contraindications; (5) Patients with complete clinical records and at least one year of follow-up.

Patients were excluded if they met any of the following conditions: (1) Patients diagnosed with cervical spondylotic radiculopathy or other types of cervical spondylosis; (2) Patients with CSM involving either a single segment or more than two segments; (3) Patients with extensive continuous ossification of the posterior longitudinal ligament (OPLL), severe focal ossification of the ligamentum flavum (OLF), or large calcified disc herniation causing spinal cord compression; (4) Patients with severe systemic diseases or coagulation disorders that rendered them unfit for surgery; (5) Patients with a history of cervical spine surgery, cervical trauma, infection, tumor, severe osteoporosis, metabolic disorders, or known allergies to implant materials; (6) Patients with incomplete follow-up data [14].

## Endo-PCCD

The paraspinal soft tissues were dissected and cleared under endoscopic visualization to fully expose the C5/6 interlaminar space. A high-speed burr was applied to initiate burring at the “V” point of the lamina to thin the laminar bone. After sufficient thinning, the lamina was grasped with lamina forceps, and the interlaminar space was opened and the surgical window gradually enlarged. This maneuver allowed clear visualization of the dural sac and the underlying spinal cord. Further enlargement of the interlaminar corridor facilitated localization of the exiting nerve root. The axillary region of the nerve root was carefully mobilized to access the herniated nucleus pulposus at the C5/6 level, which was compressing the C6 nerve root. The protruded disc material was gently grasped with straight forceps under endoscopic view. Hemostasis was achieved using a bipolar spherical radiofrequency coagulation electrode. Adhesions were meticulously separated to decompress the involved neural structures, including the spinal cord and the affected nerve root. The protruded disc fragments were completely excised. The posterior lamina was removed using a high-speed drill, and the hypertrophic ligamentum flavum was excised until the lateral edge of the contralateral dural sac was clearly visualized.

**Fig. 1:**
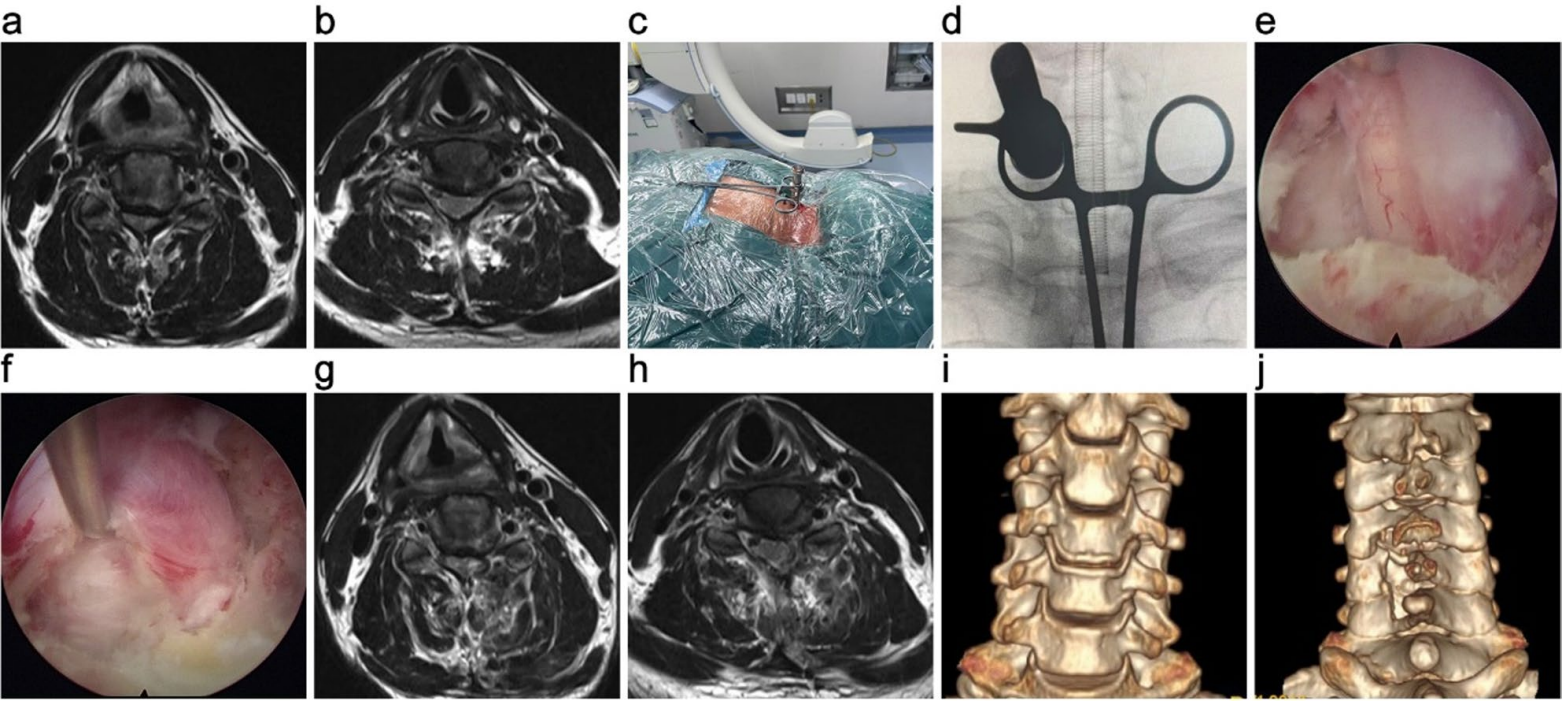
Surgical schematic and perioperative imaging manifestations of Endo-PCCD. **a-b** Preoperative MRI demonstrating spinal cord compression at the C4/5 **a** and C5/6 **b** levels; **c-d** Preoperative fluoroscopic localization; **e-f** Endoscopic decompression and spinal cord release; **g-h** Postoperative MRI showing marked relief of spinal cord compression at the C4/5 **g** and C5/6 **h** levels; **i-j** Postoperative 3D CT showing the extent of the laminotomy

A bipolar spherical radiofrequency coagulation electrode was then introduced into the intervertebral disc space, and radiofrequency ablation was performed. No active bleeding was observed under endoscopic visualization, after which the working cannula was withdrawn. The same surgical steps were subsequently repeated at the C4/5 intervertebral level (Fig. 1).

## LP

An approximately 5.0 cm longitudinal midline incision was made over the posterior cervical region. The skin, subcutaneous layer, and deep cervical fascia were incised sequentially. Subperiosteal dissection was then carried out along the spinous processes to detach the paraspinal muscles, thereby exposing the C4-C6 spinous processes and bilateral laminae, and separating the C4/5 and C5/6 intervertebral spaces. Following partial resection of the ligamentum flavum, the dural sac became clearly visible with adequate expansion.

Partial resection was performed at the lower border of the C3 spinous process, the lower border of the C6 spinous process, and the upper border of the C7 spinous process. In addition, portions of the C4-C6 spinous processes were excised. A partial cortical groove was created at the junction between the right lamina and lateral mass to serve as the hinge side (door axis), whereas a complete cortical cut was made at the corresponding location on the left side to form the open side. After completion of the osteotomy, the lamina was opened toward the right, achieving sufficient decompression of the dural sac.

**Fig. 2:**
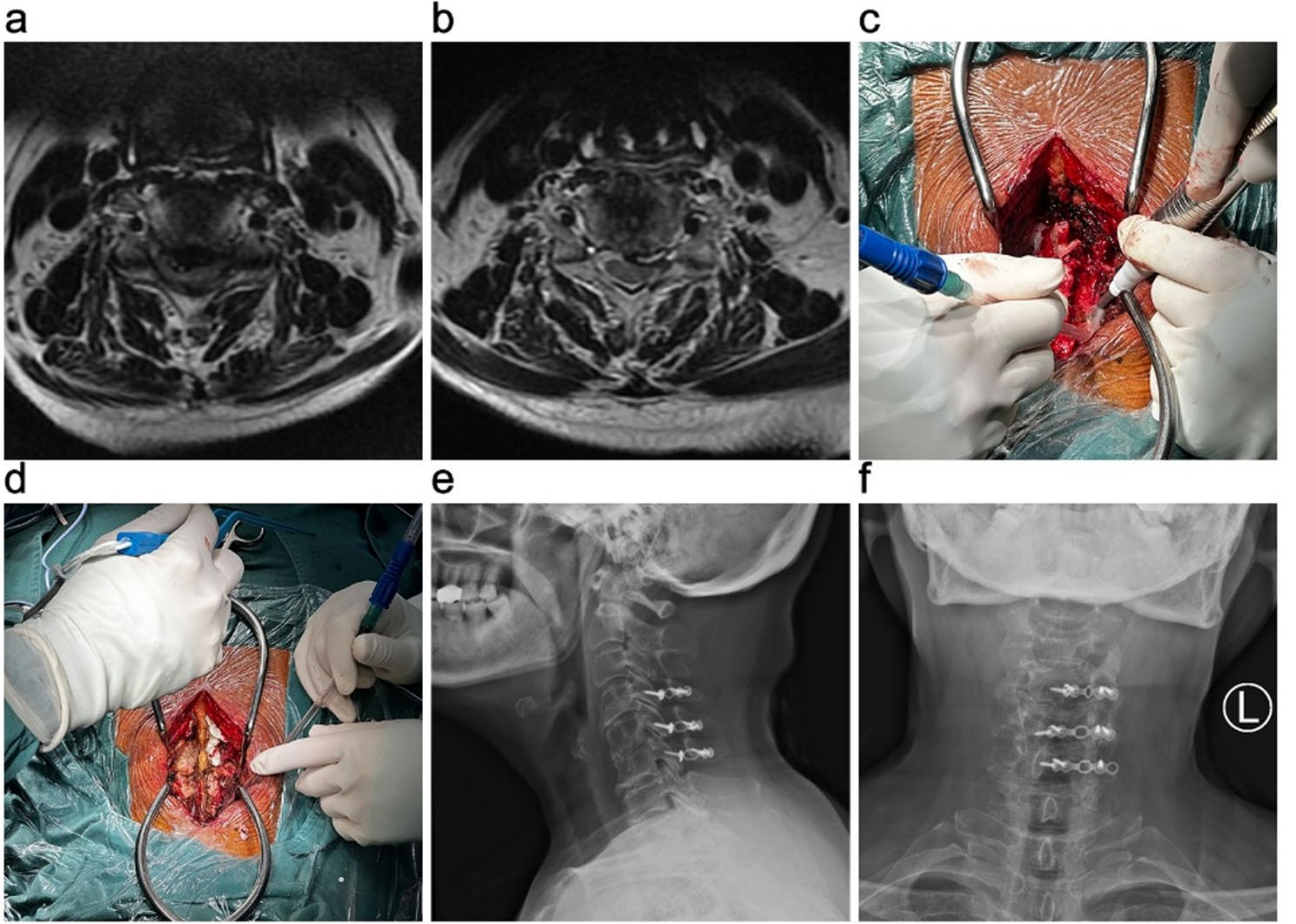
Surgical schematic and perioperative imaging manifestations of LP. **a-b** Preoperative MRI demonstrating spinal cord compression at the C4/5 and C5/6 levels; **c-d** Intraoperative schematic views of the surgical procedure; **e-f** Postoperative radiographs showing the lateral and anteroposterior views

Subsequently, lateral mass plates were implanted at the C4-C6 levels on the open side, and fixation was achieved using screws inserted into the lateral masses. The position and stability of the plates and screws were confirmed intraoperatively. Finally, the surgical wound was thoroughly irrigated with a large volume of physiological saline (Fig. 2).

## Basic parameters

Baseline demographic characteristics and perioperative parameters were recorded, including age, gender, body mass index (BMI), symptom duration, major comorbidities, operative time, and the number of surgical levels involved. Major comorbidities included hypertension, diabetes mellitus, coronary artery disease, and cerebral infarction. Preoperative and postoperative hemoglobin (Hb) levels, as well as postoperative drainage volume, were also recorded.

### Assessment of outcomes

Clinical outcomes were assessed using three validated scoring systems, the Visual Analog Scale (VAS) for pain assessment, the Japanese Orthopaedic Association (JOA) score for evaluation of neurological function before and after surgery, and the Neck Disability Index (NDI). The NDI contains 10 components covering pain intensity, personal care, lifting, reading, headaches, concentration, work, driving, sleeping, and recreation [15]. All clinical indicators were measured both preoperatively and postoperatively.

## MCID analysis

To further evaluate the clinical relevance of postoperative improvement, MCID analysis was additionally performed based on one-year follow-up outcomes. For VAS, an improvement of ≥ 2.5 points from baseline was considered clinically meaningful. For NDI, an improvement of ≥ 7.5 points was defined as achieving MCID [16]. JOA recovery rate was calculated using the Hirabayashi method as follows: recovery rate = (postoperative JOA score - preoperative JOA score) / (17 - preoperative JOA score) ×100%, and a recovery rate > 50% was considered indicative of meaningful neurological recovery [17]. The proportions of patients achieving MCID were compared between groups using the chi-square test.

## Radiographic evaluation

The dural sac area (DSA) improvement rate was calculated based on preoperative and postoperative magnetic resonance imaging (MRI), using axial T2-weighted images at the most stenotic level for measurement [18]. For patients with two decompressed segments, the mean DSA value of the two adjacent operated levels was used for analysis. The improvement rate of DSA was calculated as follows: DSA improvement rate = $\:(DS{A}_{post}-DS{A}_{pre})/DS{A}_{pre}\times\:100\mathrm{\%}$, where DSA_pre and DSA_post represent the mean preoperative and postoperative DSA values, respectively. Detailed measurement procedures are provided in the supplementary material.

Inter-observer and intra-observer reliability were evaluated using the intraclass correlation coefficient (ICC) to assess the consistency and reproducibility of DSA measurements between different observers and across repeated measurements by the same observer at a two-week interval. The ICC was calculated based on a two-way random-effects model with absolute agreement. According to commonly accepted criteria, an ICC value < 0.50 indicates poor reliability, 0.50–0.75 indicates moderate reliability, 0.75–0.90 indicates good reliability, and > 0.90 indicates excellent reliability [19].

### Perioperative and follow-up complications assessment

Perioperative and follow-up complications were systematically recorded and compared between the Endo-PCCD and LP groups. The evaluated perioperative complications included axial symptoms, C5 palsy, delayed healing of incision, cerebrospinal fluid leakage, and postoperative hematoma. Follow-up complications including neurological deterioration, restenosis, and reoperation. All complications were identified based on clinical symptoms, neurological examination findings, postoperative imaging, and medical records review. Complication assessment was performed during hospitalization and routine postoperative follow-up visits.

### Learning curve evaluation

Patients were sequentially arranged according to the chronological sequence of their surgical procedures. The learning curve was analyzed using the cumulative sum (CUSUM) method [20]. CUSUM=$\:{\sum\:}_{i=1}^{n}(Ti-T)$, where *Ti* represents the operative duration for the *i*th case, *T* denotes the average operative duration, and n indicates the case sequence number. The learning curve was performed using RStudio software (version 4.5.2), and the regression model with the greatest R^2^ value was selected as the optimal model (*P* < 0.05). The peak point of the CUSUM curve was used to distinguish the learning stage from the proficiency stage, and the corresponding case number was considered the minimum number required for surgeons to achieve procedural proficiency.

### Construction of the C2-C7 cervical spine finite element model

To construct a three-dimensional finite element model of the cervical spine, a 25-year-old healthy adult female volunteer was recruited. The participant had no history of cervical spine disease, trauma, or deformity. The study protocol complied with ethical standards, and informed consent was obtained prior to imaging acquisition.

High-resolution CT scans of the cervical spine were obtained using a 64-slice spiral CT scanner, covering C2-C7 (120 kV, 125 mA, scanning thickness 1.250 mm, slice interval 1.250 mm). A total of 149 images were obtained and exported in DICOM format.

These data were subsequently imported into Mimics 21.0 for image segmentation and three-dimensional reconstruction. The osseous structures of the cervical spine were extracted using threshold segmentation and region-growing techniques to generate the initial geometric model. The reconstructed model was subsequently imported into Geomagic studio 2021 for geometric optimization, including removal of sharp edges, spike artifacts, small fragments, and surface irregularities, followed by surface smoothing to obtain a cervical spine model with accurate anatomical structure and continuous surface.

The optimized geometry was exported as an STP file and imported into SolidWorks 2021 for detailed structural modeling. The cortical bone, cancellous bone, endplates, intervertebral discs (including annulus fibrosus and nucleus pulposus), and facet joint cartilage were constructed separately. The nucleus pulposus was assumed to occupy approximately 40% of the total intervertebral disc volume, with the remaining portion representing the annulus fibrosus. The major ligamentous structures, including the anterior longitudinal ligament, posterior longitudinal ligament, ligamentum flavum, interspinous ligament, supraspinous ligament, facet capsular ligament, and intertransverse ligament, were simulated using non-linear spring elements.

The complete C2-C7 cervical spine model containing all aforementioned structures was imported into ANSYS 2023 R1 for mesh generation and biomechanical analysis. The model was discretized using tetrahedral elements. The material properties assigned to each anatomical structure were based on previously published experimental and finite element studies, as summarized in Table 1.

**Table 1:** Material properties of each component in the cervical spine finite element model [21, 22]

Components	Yong modulus (MPa)	Poisson ration	Material model	Cross section (mm^2^)
Cortical bone	12,000	0.29	Linear elastic	–
Cancellous bone	450	0.29	Linear elastic	–
Endplate	500	0.40	Linear elastic	–
Annulus fibrosus	4.0	0.40	Hyperelastic/linear elastic	–
Nucleus pulposus	1.0	0.475	Incompressible elastic	–
Facet joint cartilage	10.4	0.40	Linear elastic	–
Anterior longitudinal ligament	7.8	0.30	Non-linear spring	6.0
Posterior longitudinal ligament	10.0	0.30	Non-linear spring	5.0
Ligamentum flavum	15.0	0.30	Non-linear spring	5.0
Interspinous ligament	10.0	0.30	Non-linear spring	10.0
Supraspinous ligament	8.0	0.30	Non-linear spring	5.0
Capsular ligament	8.0	0.30	Non-linear spring	46.0
Intertransverse ligament	10.0	0.30	Non-linear spring	2.0
Titanium plate	120,000	0.30	Linear elastic	–
Titanium screw	120,000	0.30	Linear elastic	–

### Construction of the Endo-PCCD and LP surgical finite element models

To simulate the biomechanical effects of different surgical procedures on the cervical spine, Endo-PCCD and LP surgical models were developed based on the intact model.

**Fig. 3:**
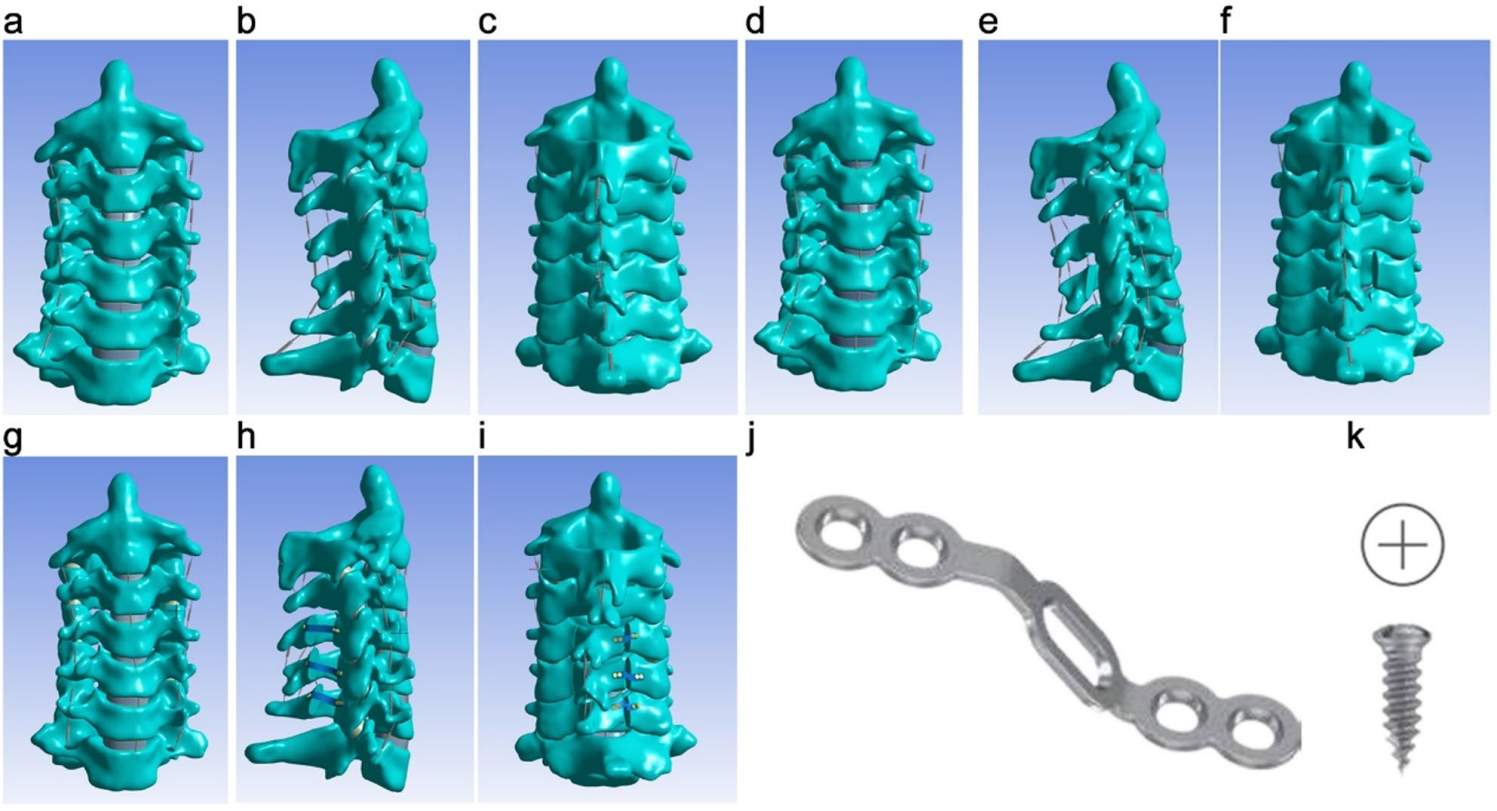
Comparison of intact and postoperative finite element models of the C2-C7 cervical spine. **a-c** Anterior, lateral, and posterior views of the intact model (control group). **d-f** Corresponding views of the model after Endo-PCCD surgery. **g-i** Corresponding views of the model after LP surgery. **j-k** Plate and screw used in the fixation construct

The intact cervical spine finite element model spanning C2-C7 served as the current model (Fig. 3a-c).

In the Endo-PCCD model, unilateral laminotomy with bilateral decompression was simulated at the C4-C6 levels. Partial resection of the lamina and ligamentum flavum was performed on the operative side. Meanwhile, the medial margin of the contralateral lamina was carefully undercut to achieve adequate bilateral spinal canal decompression. The contralateral lamina, facet joints, and facet capsular ligaments were preserved as much as possible to reflect the tissue-sparing characteristics of the Endo-PCCD procedure and its protective effect on posterior stabilizing structures (Fig. 3d-f**).**

In the LP model, open-door LP was simulated at the C4-C6 levels. A hinge was established at the lamina-lateral mass junction on one side, while a complete osteotomy was performed on the contralateral side to establish the open-door side. The lamina was then elevated toward the hinge side to expand the volume of the spinal canal. Partial resection of the spinous processes, interspinous ligaments, and ligamentum flavum was performed. A titanium plate was implanted on the open-door side for fixation to simulate the structural alterations observed in clinical LP (Fig. 3g-i**).**

The surgical internal fixation devices were constructed in SolidWorks 2021. Based on cervical anatomical characteristics and commonly used clinical implant specifications, titanium plates and screws were modeled. The plate length ranged from 8 to 14 mm with a thickness of 2.0 mm, while the screw length ranged from 5 to 12 mm and diameter was 2.0 mm (Fig. 3j-k). All implants were assigned titanium material properties and imported into ANSYS for finite element analysis.

The material properties of the remaining cervical spine components were maintained consistently in both the Endo-PCCD and LP models. These structures included the vertebral bodies, intervertebral discs, ligaments, and facet joints. Therefore, the observed biomechanical differences primarily resulted from variations in the extent of posterior structure resection and alterations in laminar morphology.

### Loading and boundary conditions

To reproduce the physiological mechanical environment of the cervical spine, appropriate loading and boundary conditions were applied to the finite element models. The inferior endplate of the C7 vertebral body was fully constrained in all degrees of freedom to represent the stability at the cervicothoracic junction. A follower load of 75 N was applied to the superior surface of the C2 vertebra to simulate the combined influence of head weight and cervical musculature. This load followed the curvature of the cervical spine to more realistically reproduce physiological axial compressive forces acting on the cervical spine. A pure moment of 2.0 N·m was applied to the C2 vertebra to simulate flexion, extension, axial rotation, and lateral bend. The same loading protocol was used for the intact model, the Endo-PCCD model, and the LP model to allow comparison of the biomechanical responses under different surgical conditions.

### Evaluation parameters

To comprehensively evaluate the biomechanical impact of Endo-PCCD and LP procedures on the cervical spine, analyses were conducted from the perspectives of stability and mobility. After applying identical boundary and loading conditions to each model, cervical spine ROM was calculated under flexion, extension, axial rotation, and lateral bend. The segmental ROM values at C2/C3, C3/C4, C4/C5, C5/C6, and C6/C7 were recorded for each model. Differences in ROM among the intact, Endo-PCCD, and LP models were then compared to evaluate the effects of the two surgical procedures on cervical kinematics were systematically analyzed.

### Statistical analysis

All statistical analyses were performed using SPSS software (version 25.0). The Shapiro-Wilk test was used to assess data normality. Continuous variables with a normal distribution were expressed as mean ± standard deviation (SD), whereas non-normally distributed data were presented as median (interquartile range). Categorical variables were analyzed using the chi-square test. Comparisons between two groups for normally distributed continuous variables were performed using independent-samples t tests, and non-normally distributed data were compared using the Mann-Whitney U test. *P* < 0.05 was considered statistically significant.

## Results

### Basic characteristics of the patients

This study included 95 patients, of whom 45 underwent Endo-PCCD and 50 underwent LP. Comparison of baseline characteristics showed no significant differences between the two groups with respect to gender, age, BMI, symptom duration, major comorbidities, or surgical levels (*P* > 0.05) (Table 2).

**Table 2:** General demographic and clinical characteristics of the two groups

Characteristics	Endo-PCCD (*n* = 45)	LP (*n* = 50)	*P* value
Age (years)	57.89 ± 12.26	59.30 ± 9.55	0.537
Gender (Male/Female)	33/12	36/14	0.884
BMI (kg/m^2^)	24.98 ± 3.41	24.63 ± 2.79	0.649
Symptom duration (months)	8.61 ± 4.84	9.20 ± 4.34	0.529
Major comorbidities			0.696
Hypertension, n (%)	11 (24.44)	10 (20.00)	
Diabetes mellitus, n (%)	10 (22.22)	5 (10.00)	
Coronary artery disease, n (%)	3 (6.67)	3 (6.00)	
Cerebral infarction, n (%)	1 (2.22)	2 (4.00)	
Surgical level			0.612
C3-C5, n (%)	6 (13.33)	4 (8.00)	
C4-C6, n (%)	19 (42.22)	25 (50.00)	
C5-C7, n (%)	20 (44.44)	21 (42.00)	

### Comparison of perioperative outcomes

The Endo-PCCD procedure required a significantly shorter operative time (113.11 ± 19.29 min) than LP (141.12 ± 21.27 min, *P* < 0.001). Furthermore, patients in the Endo-PCCD group exhibited substantially lower postoperative drainage volumes (32.27 ± 26.74 mL) compared with those in the LP group (212.06 ± 78.67 mL, *P* < 0.001). Preoperative Hb levels did not differ significantly between the two groups. However, the postoperative Hb levels were significantly higher following Endo-PCCD (134.31 ± 17.64 g/L) than after LP (126.10 ± 14.14 g/L, *P* = 0.014) (Table 3).

**Table 3:** Comparison of operative time, drainage volume, and preoperative and postoperative Hb levels between the two groups

Perioperative outcomes	Endo-PCCD	LP	*P* value
Operation time (minutes)	113.11 ± 19.29	141.12 ± 21.27	< 0.001
Drainage volume (mL)	32.27 ± 26.74	212.06 ± 78.67	< 0.001
Preoperative Hb (g/L)	138.18 ± 18.37	136.14 ± 14.34	0.546
Postoperative Hb (g/L)	134.31 ± 17.64	126.10 ± 14.14	0.014

### Comparison of clinical outcomes

Evaluation of clinical outcomes demonstrated that VAS score, JOA score, and NDI were comparable between the two groups preoperatively, at one month, and at one year postoperatively (*P* > 0.05 for all comparisons) (Table 4).

**Table 4:** Comparison of JOA score, VAS score, and NDI (%) between the two groups

Clinical outcomes	Endo-PCCD	LP	*P* value
JOA Score			
Preoperative	9.33 ± 1.57	9.76 ± 1.85	0.230
Postoperative one month	13.20 ± 0.46	13.12 ± 0.72	0.664
Postoperative one year	15.18 ± 1.05	15.02 ± 1.22	0.504
VAS Score			
Preoperative	6.04 ± 0.95	6.16 ± 1.27	0.620
Postoperative one month	3.64 ± 1.19	3.98 ± 1.25	0.185
Postoperative one year	2.13 ± 1.34	1.72 ± 1.05	0.096
NDI (%)			
Preoperative	34.58 ± 6.55	35.60 ± 6.44	0.445
Postoperative one month	20.47 ± 5.91	21.60 ± 6.21	0.366
Postoperative one year	11.91 ± 4.80	12.44 ± 4.43	0.578

### Comparison of MCID achievement

At the one-year follow-up, the proportions of patients achieving MCID for VAS and NDI, as well as meaningful JOA recovery, were comparable between the two groups (*P* > 0.05 for all comparisons), indicating similar clinically meaningful improvement after surgery (Table 5).

**Table 5:** Comparison of MCID achievement rates at one-year follow-up between the two groups

Outcome	Endo-PCCD (*n* = 45)	LP (*n* = 50)	*P* value
VAS MCID achieved, n (%)	33 (73.33)	39 (78.00)	0.596
NDI MCID achieved, n (%)	36 (80.00)	43 (86.00)	0.435
JOA recovery > 50%, n (%)	35 (77.78)	38 (76.00)	0.838

#### Comparison of DSA improvement between the two groups

For the C3-C5 segment, the average improvement rate of the DSA was 27.35 ± 5.62% in the Endo-PCCD group and 55.55 ± 36.56% in the LP group, with no statistically significant difference between the two procedures (*P* = 0.092). In the C4-C6 segment, the postoperative improvement rate of DSA was 45.62 ± 28.40% in the Endo-PCCD group and 58.01 ± 18.53% in the LP group, without reaching statistical significance (*P* = 0.090). For the C5-C7 segment, the improvement rate of DSA was 45.92 ± 8.16% in the Endo-PCCD group, whereas the LP group showed a significantly higher value of 60.04 ± 12.41% (*P* < 0.001) (Table 6). The inter-observer, Observer 1 intra-observer and Observer 2 intra-observer ICC values for DSA measurements were 0.987 (0.981–0.991), 0.984 (0.954–0.992) and 0.971 (0.815–0.989), respectively, indicating excellent measurement reliability (**Supplementary Table 1**).

**Table 6:** Comparison of postoperative improvement rates of the DSA between the two groups

Components	Endo-PCCD	LP	*P* value
Postoperative DSA improvement rate at C3-C5 (%)	27.35 ± 5.62	55.55 ± 36.56	0.092
Postoperative DSA improvement rate at C4-C6 (%)	45.62 ± 28.40	58.01 ± 18.53	0.090
Postoperative DSA improvement rate at C5-C7 (%)	45.92 ± 8.16	60.04 ± 12.41	< 0.001

### Perioperative and follow-up complications

No severe complications, such as permanent neurological injury or deep infection, were observed in either group. Perioperative complications including axial symptoms, C5 palsy, delayed incision healing, cerebrospinal fluid leakage, and postoperative hematoma, were comparable between the Endo-PCCD and LP groups, with no statistically significant differences observed (*P* > 0.05) (Table 7). All complications were relieved after symptomatic and conservative treatment. No patient experienced neurological deterioration, restenosis, or required revision surgery during the follow-up period.

**Table 7:** Comparison of perioperative and follow-up complications between the two groups

Characteristics	Endo-PCCD (*n* = 45)	LP (*n* = 50)	*P* value
Axial symptoms, n (%)	4 (8.89)	10 (20.00)	0.127
C5 palsy, n (%)	2 (4.44)	1 (2.00)	0.496
Delayed healing of incision, n (%)	0 (0.00)	1 (2.00)	0.340
Cerebrospinal fluid leakage, n (%)	1 (2.22)	1 (2.00)	0.940
Postoperative hematoma, n (%)	0 (0.00)	1 (2.00)	0.340

#### Learning curve analysis

**Fig. 4:**
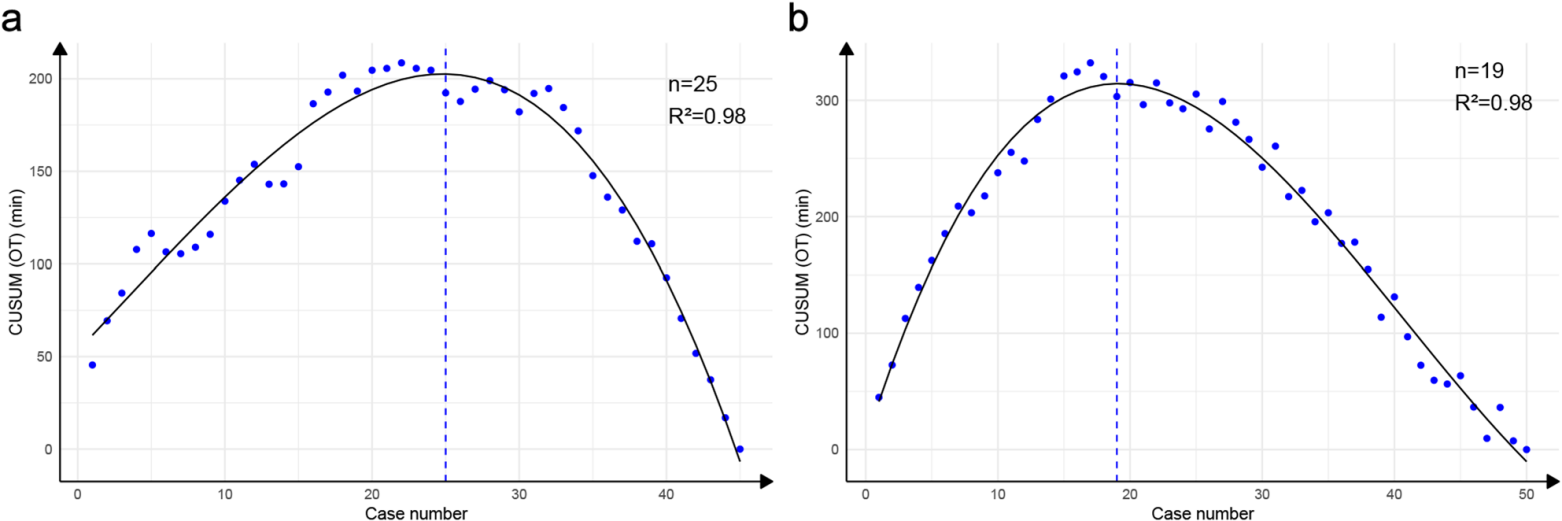
Learning curves based on CUSUM analysis. **a** Endo-PCCD group. **b** LP group

The fitted CUSUM learning curve for the Endo-PCCD group was described by the equation y = − 0.00604378 × ^3^ + 0.05766367 × ^2^ + 8.310725x + 5.683639 (R^2^ = 0.98). For the LP group, the fitted equation was y = 0.01003254 × ^3^ − 1.225635 × ^2^ + 35.86208x + 5.683639 (R^2^ = 0.98) (Fig. 4). The learning curve peaked at 25 cases in the Endo-PCCD group and at 19 cases in the LP group. Based on the peak points of the CUSUM curves, the thresholds for achieving surgical proficiency were identified as 25 cases for the Endo-PCCD group and 19 cases for the LP group.

#### Validation of a complete cervical spine finite element model

**Fig. 5:**
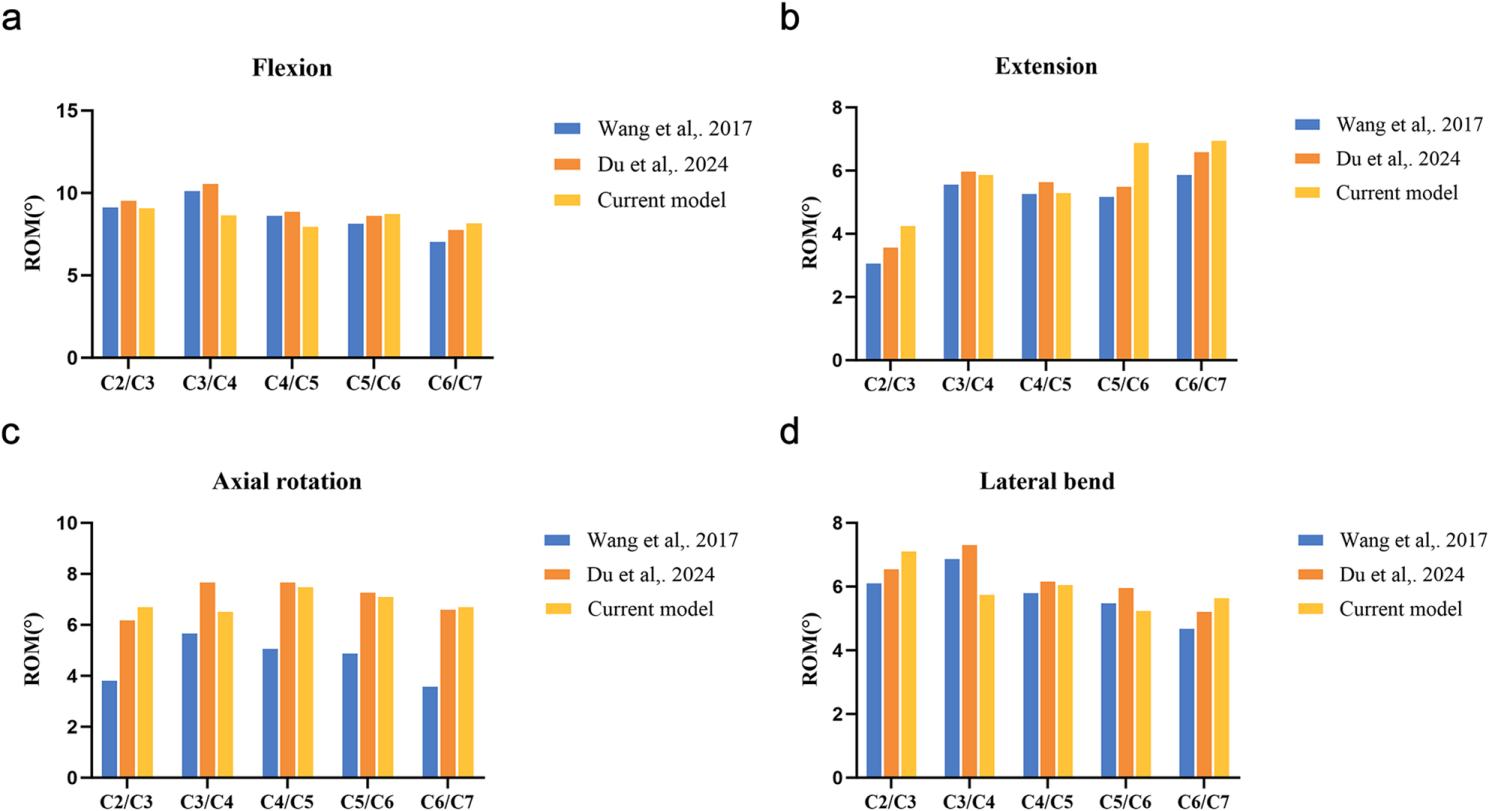
Comparison of the ROM between the current finite element model and previously published studies. **a** Flexion; **b** Extension; **c** Axial rotation; **d** Lateral bend

Model validation was performed by comparing the predicted ROM of the intact cervical spine model under flexion, extension, axial rotation, and lateral bend with values reported in previous studies. The results demonstrated that the overall motion patterns predicted by the present model were highly consistent with those reported in prior finite element investigations [23, 24]. Moreover, the segmental ROM values were all within the physiological ranges documented in the literature (Fig. 5). These findings confirm that the present finite element model provides reliable biomechanical predictions and can be used for subsequent comparative analyses.

#### Comparison of segmental ROM between LP and Endo-PCCD models

**Fig. 6:**
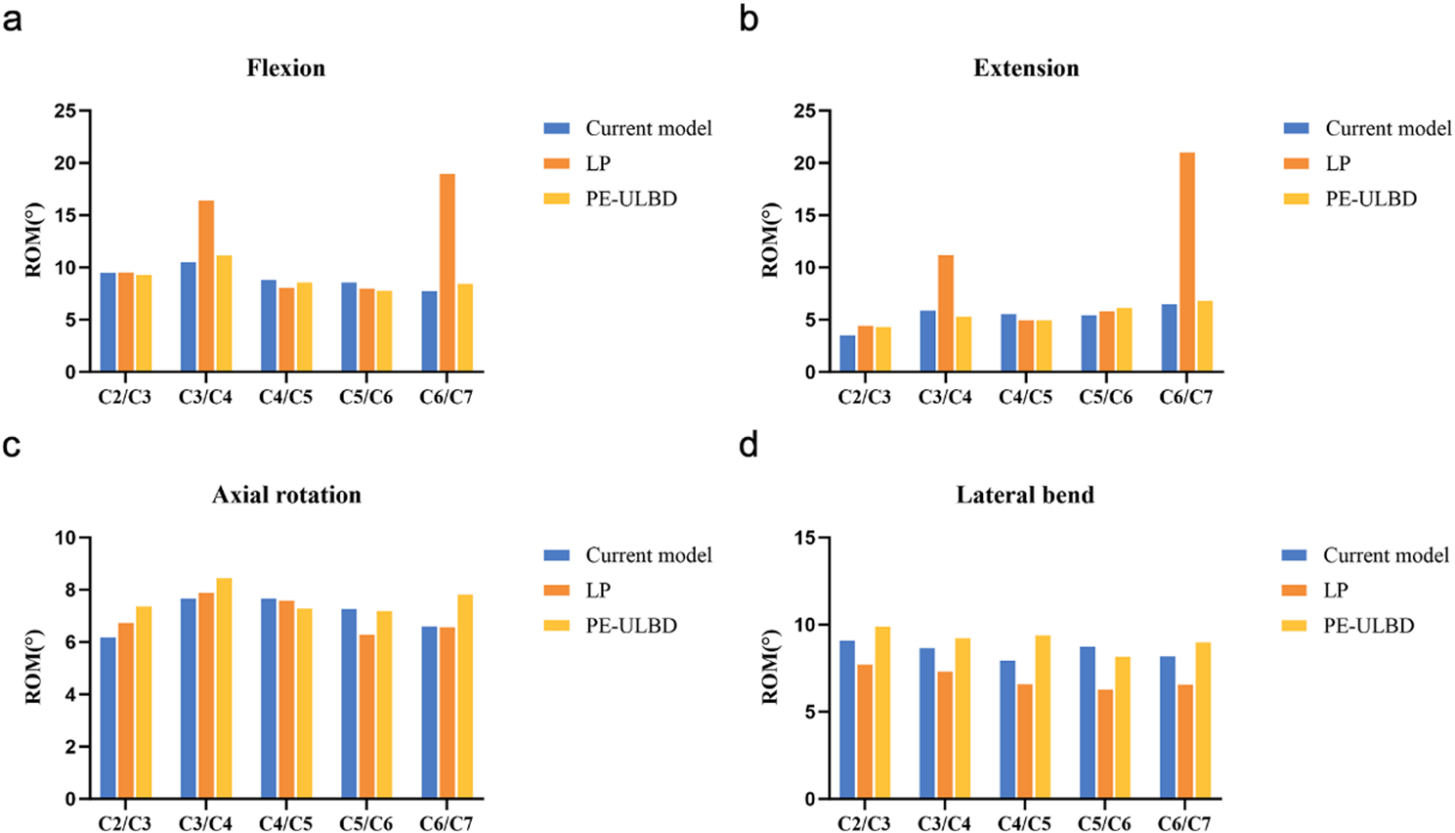
Comparison of segmental ROM of the cervical spine between the LP and Endo-PCCD models under different loading conditions. **a** Flexion; **b** Extension; **c** Axial rotation; **d** Lateral bend

The segmental ROM was compared between the two surgical models. The results showed that, relative to the Endo-PCCD model, the LP model exhibited an overall increasing trend in segmental ROM under flexion and extension, with the most pronounced increases observed at the C3/C4 and C6/C7 levels. In contrast, the Endo-PCCD model demonstrated relatively smaller changes in ROM during flexion and extension, and its overall segmental motion pattern was more consistent with that of the intact model (Fig. 6a-b). Furthermore, under axial rotation and lateral bend conditions, both surgical models demonstrated ROM values comparable to those of the intact model, with no obvious differences observed (Fig. 6c-d**).** These findings indicate that the LP model results in greater segmental mobility under flexion and extension, whereas the Endo-PCCD model better preserves motion characteristics similar to the intact condition, reflecting a biomechanical behavior closer to the physiological state.

## Discussion

LP is performed through a midline posterior cervical incision that exposes the target segments and allows partial laminar removal for decompression (Fig. 2). The procedure enlarges the spinal canal to relieve spinal cord compression while aiming to preserve cervical stability and reduce postoperative scar formation associated with neurological deterioration [25]. In contrast, Endo-PCCD is performed via a posterior interlaminar approach using a working channel, which may reduce disruption to the facet joint and preserves the integrity of the posterior spinal elements, thereby may theoretically contribute to relative preservation of cervical biomechanical stability [26]. Intuitively, compared with LP, Endo-PCCD is performed through a smaller surgical corridor and may be associated with less soft tissue disruption.

Surgical duration and intraoperative blood loss are important considerations in spinal surgery. Monetta et al. reported that prolonged operative duration is closely related to a higher incidence of adverse events, highlighting the importance of reducing surgical duration to improve patient safety [27]. Excessive intraoperative blood loss may also increase the need for blood transfusions, which carries risks such as postoperative infection, infectious disease transmission, and acute hemolytic reactions [28]. In the current study, the surgical duration was significantly shorter in the Endo-PCCD group than in the LP group, possibly because LP requires broader anatomical exposure and longer time needed for suturing postoperatively. Preoperative Hb levels were comparable between groups; however, postoperative Hb levels were significantly higher in patients treated with Endo-PCCD. In contrast, postoperative drainage volume was markedly greater in the LP group than in the Endo-PCCD group, indirectly suggesting relatively increased perioperative blood loss in patients undergoing LP. One possible explanation is that LP generally requires broader soft tissue exposure and more extensive posterior element manipulation, which may contribute to increased bleeding [29]. However, because direct measurements of tissue injury or intraoperative vascular damage were not included in the present study, these interpretations should be considered exploratory.

The present study showed that LP achieved a greater postoperative DSA improvement rate than Endo-PCCD. Nevertheless, adequate decompression was achieved in both groups, as evidenced by significant postoperative improvements in JOA and VAS scores compared with preoperative values. Importantly, postoperative VAS scores, JOA scores, complication rates, and patient satisfaction were comparable between the two groups. In addition to statistical comparisons, MCID analysis demonstrated comparable rates of clinically meaningful recovery between the two groups, further supporting the similar clinical effectiveness observed in conventional statistical analyses. Similar findings have been reported previously. Hermansen et al. reported that in patients with lumbar spinal stenosis, the perioperative enlargement of the dural sac cross-sectional area was not significantly correlated with clinical parameters or symptom improvement during either early postoperative evaluation or long-term follow-up [30]. These findings suggest that greater radiological decompression may not necessarily translate into superior short-term clinical outcomes. Endoscopic spinal surgery is increasingly favored due to its minimally invasive characteristics, including smaller incisions, reduced postoperative pain, and shorter hospital stays; however, it is also associated with a relatively steep learning curve [31]. Sun et al. reported that novice surgeons required approximately 23 cases to achieve technical proficiency in PECD [32]. In the present study, surgical proficiency was achieved after approximately 19 cases in the LP group and 25 cases in the Endo-PCCD group. The longer learning curve observed for Endo-PCCD may be attributed to several factors. First, the procedure is performed through a limited surgical corridor and requires precise endoscopic manipulation and instrument handling. Second, the working space is relatively restricted, with visualization relying entirely on endoscopic imaging, which demands accurate endoscope positioning and coordinated instrument movements. In contrast, LP provides a wider exposure, allowing for more direct visualization and easier manipulation. Third, Endo-PCCD requires detailed preoperative imaging evaluation to accurately identify lesion characteristics and adjacent anatomical structures, placing greater demands on surgical experience. In contrast, LP involves broader exposure and relatively straightforward anatomical identification, resulting in simpler preoperative preparation.

Biomechanical evaluation using finite element analysis demonstrated distinct differences in segmental ROM patterns between the two surgical techniques. Since the finite element model was established from a healthy volunteer under standardized conditions, the present findings should primarily be interpreted as relative biomechanical comparisons rather than direct representations of postoperative clinical behavior.

The LP model demonstrated increased segmental ROM under flexion and extension, particularly at the C3/C4 and C6/C7 levels [33]. Clinically, increased postoperative ROM does not necessarily indicate pathological instability, as preservation of cervical motion is one of the intended characteristics of LP procedures. In some cases, restoration or maintenance of mobility after decompression may reflect recovery of segmental motion following relief of chronic spinal cord compression. However, relatively greater segmental motion may also indicate altered postoperative biomechanical characteristics of the cervical spine, especially following surgical alteration of posterior cervical structures [34]. Previous biomechanical studies have indicated that excessive segmental motion may influence load distribution and local mechanical environments surrounding neural structures, which could potentially contribute to long-term degenerative changes or delayed neurological symptoms [33, 34]. Therefore, the increased ROM observed in the LP model may reflect a partial restoration of cervical motion following decompression, while also suggesting postoperative remodeling of the segmental biomechanical environment.

In contrast, the Endo-PCCD model demonstrated segmental motion patterns that were closer to those of the intact cervical spine. From a biomechanical perspective, preservation of physiological motion patterns may contribute to maintaining cervical motion characteristics closer to those of the intact spine. This may be related to the greater preservation of posterior structures in Endo-PCCD, including the spinous processes, interspinous ligaments, and facet joint complex. Maintenance of these structures may contribute to preserving the physiological instantaneous center of rotation and overall biomechanical coordination of the cervical spine [35]. Although the present finite element analysis did not directly evaluate neural stress or long-term clinical outcomes, these findings suggest that Endo-PCCD may better preserve postoperative physiological biomechanics. Nevertheless, the clinical significance of these biomechanical differences still requires confirmation through longer-term prospective clinical studies.

Despite the promising findings, several limitations should be noted. First, the study was conducted as a single-center retrospective analysis with a relatively limited sample size, which may introduce potential selection bias and restrict the external applicability of the results. Future multicenter prospective investigations with larger cohorts are necessary to confirm these observations. Second, the finite element model was established based on imaging data from a single healthy volunteer, which may limit the generalizability of the biomechanical findings, particularly for patients with cervical degenerative pathology. In addition, the model involved certain simplifications of physiological conditions, without incorporating the dynamic effects of cervical musculature, individual anatomical variations, or neural tissues. Therefore, the evaluation of neural stress was inferred indirectly from segmental kinematic changes, and the current model may not fully reflect the actual postoperative biomechanical environment. Third, the learning curve analysis was primarily based on operative time, which may not comprehensively reflect surgical proficiency. Additional indicators, including perioperative complications and postoperative clinical outcomes, should be incorporated in future studies to provide a more comprehensive evaluation of surgical mastery. In addition, the relatively short follow-up duration may not fully capture long-term outcomes such as adjacent segment degeneration, delayed instability, or other late complications, which warrants further investigation with extended follow-up.

## Conclusion

In conclusion, by integrating clinical outcomes with finite element analysis, this study provides a comprehensive evaluation of the perioperative performance and biomechanical impact of two surgical strategies for cervical spondylotic myelopathy. Endo-PCCD achieved clinical outcomes comparable to LP while demonstrating a potential trend toward reduced surgical invasiveness and perioperative burden. Importantly, it preserved cervical motion patterns more closely resembling the intact physiological state, suggesting a potential advantage in maintaining spinal stability and function. Although LP may offer greater decompression at specific levels, Endo-PCCD represents a less invasive and motion-preserving alternative, which may be particularly beneficial for selected patients requiring targeted decompression. These findings support the clinical applicability of Endo-PCCD and highlight its potential role in optimizing the balance between effective decompression and biomechanical preservation.

## Supplementary Information

Below is the link to the electronic supplementary material.

Supplementary Material 1

## Data Availability

The raw data are not publicly available due to patient privacy, but may be obtained from the corresponding author upon reasonable request and subject to approval by the institutional ethics committee.
