# Supplementary material for: Endoscopic posterior cervical canal decompression versus laminoplasty for two-level cervical spondylotic myelopathy: clinical outcomes and finite element analysis
Source: J Orthop Surg Res. 2026 Jun 8;21:494. doi: 10.1186/s13018-026-07000-1 (PMC13471376; doi:10.1186/s13018-026-07000-1)
Supplement: Supplementary file 1 [file 13018_2026_7000_MOESM1_ESM.docx]

**Supplementary Methods**

**Calculation of the improvement rate of dural sac area (DSA)**

Measurements were performed using axial T2-weighted MRI images at the most stenotic level. Dural sac area (DSA) was quantified using VISIONPACS software. For patients undergoing two-level decompression, the average DSA value of the two adjacent decompressed segments was used to represent the overall decompression effect. All measurements were independently performed by two experienced observers who were blinded to the patients’ clinical outcomes and to each other’s measurements. To assess intra-observer reliability, one observer repeated all measurements after a 2-week interval. The mean value of the measurements obtained by the two observers was used for the final analysis.

Taking C4-C6 Endo-PCCD as an example, the improvement rate of the DSA was calculated as follows:

The DSA was measured at the most stenotic levels of C4/C5 and C5/C6 on preoperative imaging. Specifically, the DSA at C4/C5 was defined as a_1_ (**Supplementary Fig. 1a**), and the DSA at C5/C6 was defined as a_2_ (**Supplementary Fig. 1b**). Postoperatively, the DSA was measured at the corresponding levels of C4/C5 and C5/C6. The postoperative DSA at C4/C5 was defined as b_1_ (**Supplementary Fig. 1c**), and that at C5/C6 was defined as b_2_ (**Supplementary Fig. 1d**).

The mean preoperative DSA was calculated as: $DSA_{pre}=(a_{1}+a_{2})/2$, and the mean postoperative DSA was calculated as:$DSA_{post}=(b_{1}+b_{2})/2$. The improvement rate of DSA was calculated using the following formula: $\text{Improvement rate of DSA}=\frac{DSA_{post}-DSA_{pre}}{DSA_{pre}}\times100\%$.

**Supplementary Fig. 1** Axial T2-weighted MRI images illustrating the measurement of the dural sac area (DSA) at C4/C5 and C5/C6 levels in a representative patient undergoing C4-C6 Endo-PCCD. **a-b** Preoperative DSA measured at the most stenotic levels of C4/C5 and C5/C6. **c-d** Postoperative DSA measured at the corresponding levels of C4/C5 and C5/C6

**Supplementary Results**

**Supplementary Table 1** Inter-observer and intra-observer reliability analysis for DSA measurements

| Reliability analysis | ICC (95% CI) | Grade |
| --- | --- | --- |
| Inter-observer reliability (n = 95, k = 2) | 0.987 (0.981-0.991) | Excellent |
| Observer 1 intra-observer reliability (n = 95, k = 2) | 0.984 (0.954-0.992) | Excellent |
| Observer 2 intra-observer reliability (n = 95, k = 2) | 0.971 (0.815-0.989) | Excellent |

Note: Inter-observer reliability was calculated based on the first measurements performed independently by the two observers. Intra-observer reliability was assessed using repeated measurements performed by the same observer at a 2-week interval.
